# Enhanced detection of low-level viremia by cobas 5800/6800 testing systems: a multi-center evaluation and clinical calibration study in China

**DOI:** 10.3389/fcimb.2026.1864911

**Published:** 2026-07-17

**Authors:** Yu Wang, Jianjian Li, Pinliang Pan, Jiafa Liu, Xin Zhang, Di Han, Meilin Wang, Tianshu Li, Cong Jin

**Affiliations:** 1 National Key Laboratory of Intelligent Tracking and Forecasting for Infectious Diseases, National Center for AIDS/STD Control and Prevention, Chinese Center for Disease Control and Prevention, Beijing, China; 2 Department of Clinical Laboratory, Yunnan Provincial Infectious Disease Hospital, Kunming, China

**Keywords:** China, HIV-1 viral load testing, low-level viremia, multi-center evaluation, performance evaluation, viral suppression

## Abstract

**Introduction:**

With the advent of more sensitive HIV-1 viral load (VL) testing systems, a greater number of low VL samples can be identified. However, for some patients who were previously virally suppressed, the new testing system may yield discordant results, posing challenges for clinical decision-making. This study systematically evaluated and compared the performance of the cobas 5800/6800 testing systems and the cobas AmpliPrep/cobas TaqMan (CAP/CTM) testing systems.

**Methods:**

The performance of the cobas 5800/6800 and CAP/CTM systems was assessed in terms of quantitative accuracy, precision, limits of detection, linearity, and clinical performance. A multi-center evaluation involving 18 sub-centers was conducted, and correlation and agreement analyses were performed on the quantitative results between the systems. The VL distribution among 1,486 patients in Yunnan Province was also analyzed to evaluate the impact of switching to the cobas 6800 system.

**Results:**

All the systems achieved a quantitative bias within 0.25 log and intra-run CV% below 5% at 10,000 and 1,000 IU/mL, while CAP/CTM demonstrated a CV% slightly exceeding 5% at 100 IU/mL. The limits of detection (cobas 5800/6800: 24 IU/mL; CAP/CTM: 30 IU/mL) matched manufacturer specifications, with all systems showing excellent linearity (R²=0.9965–0.9990) and subtype coverage. Correlation analysis showed strong associations (R²>0.90), yet the quantitative results of cobas 5800 and cobas 6800 showed a modestly higher, but consistent bias compared to CAP/CTM. Analysis of the VL distribution showed that switching to the cobas 6800 testing system significantly increased the proportion of VL between 20–200 copies/mL; however, no significant difference was observed after calculation using the correlation coefficient obtained from the multi-center evaluation.

**Conclusion:**

In summary, both the legacy and new testing systems exhibited excellent performance in detecting HIV-1 subtypes prevalent in China. This study provides reliable data to enable more accurate assessment of viral suppression efficacy during the transition period.

## 1 Introduction

Given its higher sensitivity and specificity, resulting in a shorter window period, HIV-1 nucleic acid testing (NAT) has become a well-established clinical tool, playing an essential role in both diagnosing HIV-1 infection and monitoring the efficacy of antiretroviral therapy (ART) (Zhao et al., 2019; National Center for HIV/AIDS, Viral Hepatitis, STD, and TB Prevention (US), 2023; Williams et al., 2023; Chinese Center for Disease Control and Prevention, 2025). Quantitative HIV-1 RNA testing provides a numerical viral load (VL) value, which is essential for monitoring therapeutic efficacy, allowing for timely detection of potential antiretroviral treatment failure and the adjustment of treatment regimens.

A lack of VL suppression after the initiation of ART indicates the need for clinical interventions, including medication adherence counseling, drug resistance testing, or adjustment of the treatment regimen. Meanwhile, given the growing number of HIV-1 low-level viremia (LLV) (Zhao et al., 2025), both China and other international organizations have adjusted the criteria for viral suppression to 50 copies/mL (AIDS and Hepatitis C Professional Group, Society of Infectious Diseases, Chinese Medical Association and Chinese Center for Disease Control and Prevention, 2021; World Health Organization, 2021; Department of Health and Human Services (US), 2025; European AIDS Clinical Society, 2025). Therefore, employing highly sensitive and precise VL testing methods is crucial, as they facilitate the earliest possible detection of LLV and/or treatment failure.

In recent years, advancements in HIV-1 NAT technology have enhanced the sensitivity and accuracy of HIV-1 VL testing. Among these, Roche’s HIV-1 VL testing systems have undergone performance upgrades to enhance compatibility and quantitative accuracy. Key technical improvements in the new-generation systems (cobas 6800/8800 and cobas 5800, Roche, Switzerland) include enhanced template purification, optimized polymerase efficiency, an additional *LTR* primer for broader subtype coverage, and a non-competitive internal control. These technological improvements have enhanced the sensitivity of the testing systems, enabling the detection of more HIV-1 RNA and better identification of LLV. This facilitates more precise clinical evaluation of disease progression and treatment efficiency in HIV-1 infections.

However, this shift in analytical sensitivity creates a universal challenge: patients with stable virological control may appear to have detectable or elevated VL solely due to the enhanced detection capability of the new systems, rather than true treatment failure. Consequently, a direct replacement of testing systems without proper interpretation tools risks artificially depressing the reported viral suppression rates, undermining progress toward the UNAIDS 95-95–95 targets. Previous studies have shown the new-generation testing systems yield modestly higher quantitative results (Wirden et al., 2017; Tan et al., 2018; Carmona et al., 2022; Tschäpe et al., 2022; Wirden et al., 2022), which may cause inconsistent viral suppression assessment during the transition, posing challenges for clinical decision-making and the achievement of the third 95% target. According to data from the external quality assessment organized by the National HIV Reference Laboratory, by the end of 2024, CAP/CTM accounted for approximately 35% of all participating laboratories. The direct replacement of the testing system is very likely to lead to a decreased proportion of HIV infections on treatment achieving viral suppression. Therefore, it is imperative to conduct a systematic evaluation to understand the performance of the new-generation testing systems for HIV-1 subtypes prevalent in China.

In this study, we systematically evaluated and compared the analytical performance of the new cobas 5800/6800 testing system and CAP/CTM system. Through a multi-center evaluation, we derived a conversion factor for VL results between the new and old systems. This conversion factor was then applied to clinical testing data from Yunnan Province to provide a comparable assessment approach during the transition period between the old and new systems, enabling healthcare service systems such as disease control centers and hospitals to better assess the effectiveness of viral suppression.

## 2 Materials and methods

### 2.1 Performance evaluation

Between January 15, 2025 and February 25, 2025, we completed the preparation of the performance evaluation panel and the multi-center evaluation panel, as well as the performance evaluation of the cobas 5800, cobas 6800, and CAP/CTM test systems, at the National Center for AIDS/STD Control and Prevention, Chinese Center for Disease Control and Prevention.

Guidelines (Clinical and Laboratory Standards Institute, 2012; Clinical and Laboratory Standards Institute, 2014; World Health Organization, 2017a; World Health Organization, 2017b; China National Accreditation Service for Conformity Assessment, 2019a; China National Accreditation Service for Conformity Assessment, 2019b) for evaluating the performance of quantitative testing were referenced to assess the precision, limit of detection (LOD), linearity, and clinical performance of the cobas HIV-1 on the cobas 5800/6800 and HIV-1 Test v2.0 on the CAP/CTM, as well as the correlation and agreement between these testing systems.

#### 2.1.1 Quantitative accuracy and precision analysis

The National Standard for HIV-1 RNA quantification (130,000 IU/mL; Lot: 220021-20160101, National Institutes for Food and Drug Control, Beijing, China) were diluted to three concentrations: 10,000 IU/mL, 1,000 IU/mL and 100 IU/mL using HIV-1 negative plasma. In each trial run, the standards were tested in five replicates, with a total of three independent runs conducted in separated days. To evaluate the quantitative accuracy, the quantitative bias between the mean measured values and the theoretical values for each concentration were calculated (Clinical and Laboratory Standards Institute, 2014; World Health Organization, 2017a; World Health Organization, 2017b; China National Accreditation Service for Conformity Assessment, 2019a; China National Accreditation Service for Conformity Assessment, 2019b).

#### 2.1.2 LOD

The National Standard for HIV-1 RNA quantification (130,000 IU/mL; Lot: 220021-20160101, National Institutes for Food and Drug Control, Beijing, China) was subjected to a two-fold serial dilution to five concentrations: 320 IU/mL, 160 IU/mL, 80 IU/mL, 40 IU/mL, and 20 IU/mL, with each concentration tested in 20 replicates. A Probit regression was performed, and the predicted value at a 95% positive detection rate was calculated as the LOD of each system (Clinical and Laboratory Standards Institute, 2012; World Health Organization, 2017a; World Health Organization, 2017b; China National Accreditation Service for Conformity Assessment, 2019a; China National Accreditation Service for Conformity Assessment, 2019b).

#### 2.1.3 Linearity analysis

Five samples were prepared by ten-fold serial dilution of an HIV-1 subtype CRF_01 AE sample (6.74 log10 IU/mL), as determined by the CAP/CTM. For each system, the five samples were tested in triplicate, and a standard curve was constructed based on the mean results. The linear regression equation and linear correlation coefficient were then calculated for each system (World Health Organization, 2017a; World Health Organization, 2017b; China National Accreditation Service for Conformity Assessment, 2019a; China National Accreditation Service for Conformity Assessment, 2019b).

#### 2.1.4 Clinical performance

The sensitivity of HIV-1 VL kits was evaluated by twenty-eight HIV-1 positive plasma samples, including 20 samples with VL ≥ 5000 IU/mL and 8 samples with VL < 5000 IU/mL (5 samples with VL between 1000–5000 IU/mL; 2 samples with VL between 200–1000 and 1 sample with VL < 200), as determined by CAP/CTM. These samples covered the five most prevalent HIV-1 subtypes in China: CRF_07BC, CRF_08BC, CRF_01AE, CRF_5501B and B. The specificity was evaluated by twenty-nine HIV-1 negative samples, including 3 hepatitis C virus (HCV) positive samples, 3 hepatitis B virus (HBV) positive samples, and 3 treponema pallidum (Tp) positive samples.

### 2.2 Multi-center evaluation

The multi-center evaluation panel consisted of dilutions of viral culture supernatants from the five most prevalent HIV-1 subtypes in China and one HIV-1 negative plasma sample (**Table 1**). After preliminary quantification by cobas 5800, the viral culture supernatants were subjected to gradient dilution using HIV-1 negative plasma, with each subtype comprising seven concentration levels.

**Table 1:** Composition of the multi-center evaluation panel.

Viral loads	Number of samples
>5,000 IU/mL	1 sample per subtype
1,000 - 5,000 IU/mL	1 sample per subtype
200 - 1,000 IU/mL	1 sample per subtype
50–200 IU/mL	1 sample per subtype
< 50 IU/mL	3 samples per subtype
HIV-1 negative sample	1 sample

To achieve precise quantification of the HIV-1 positive samples in the panel, the 4th HIV-1 International Standard (WHO-IS NIBSC code: 16/194, VL: 5.10 log10 IU/mL, NIBSC Hertfordshire UK) was subjected to a five-fold serial dilution to generate five concentrations of standards. The diluted standard series and the samples to be quantified were tested simultaneously on the cobas 5800 system, with three replicates each for both the standards and samples to calculate the mean values. A linear regression equation was established based on the theoretical values and the measured mean values of each standard concentration. Sample VL were then quantified against this standard curve.

We included 18 laboratories in China to evaluate the panel between March 1, 2025 and March 31, 2025, with 6 laboratories each capable to perform cobas 6800, cobas 5800, and CAP/CTM VL testing (**Table 2**). We calculated the mean value of the test results to analyze the correlation and the agreement between each pair of the testing systems. Furthermore, we analyzed and compared the inter-laboratory precision and the trend of VL distribution of each system.

**Table 2:** Laboratories participating in the multi-center evaluation.

Cobas 6800	Cobas 5800	CAP/CTM
Yunnan Provincial Hospital of Infectious Disease	Peking Union Medical College Hospital	Peking Union Medical College Hospital
Chest Hospital of Guangxi Zhuang Autonomous Region	Yunnan Province Yuanyang County People’s Hospital	Sichuan CDC
Honghe Prefecture First People’s Hospital of Yunnan Province	Nanyang CDC, Henan Province	Henan CDC
Bazhong CDC, Sichuan Province	The Third People’s Hospital of Changzhou, Jiangsu Province	Jiangsu CDC
Ningbo CDC, Zhejiang Province	Shandong CDC	Shandong CDC
Yubei CDC, Chongqing Municipality	Chongqing CDC	Chongqing CDC

### 2.3 Clinical application evaluation

We retrospectively collected HIV-1 VL testing records from Yunnan Provincial Hospital of Infectious Disease between January 2021 and December 2025. All patients who met the following criteria were included consecutively (1): had at least one VL test result before March 10, 2023 (using CAP/CTM) and at least one after that date (using cobas 6800); (2) did not change their ART regimen during the observation period; (3) had no missing records for the required time points. A total of 1,486 patients met these criteria.

To mitigate the systematic bias introduced by the transition and to enable longitudinal comparability of viral suppression rates across the systems transition, we also used the correlation equation obtained from the multi-center evaluation between the cobas 6800 and CAP/CTM systems to convert the VL of these patients. By calculating the proportion of patients between the scopes of clinical decision points after conversion, we further evaluated the potential clinical utility of this conversion coefficient during the transition period between the old and new testing systems.

### 2.4 Statistical analysis

HIV-1 RNA values were converted to IU/mL and then transformed to log_10_ IU/mL. LOD was calculated via Probit regression (SPSS v21.0, Armonk, USA), linear regression and Bland-Altman analysis (GraphPad Prism 8.0, Boston MA, USA) were used to assess correlation and agreement between systems.

## 3 Results

### 3.1 Performance evaluation

#### 3.1.1 Quantitative accuracy and precision analysis of cobas 6800, cobas 5800 and CAP/CTM

All three systems showed quantitative biases within 0.25 log across all tested concentrations, with cobas 6800 demonstrating the smallest deviation. Additionally, cobas 6800 and cobas 5800 maintained intra-run CV%<5% when detecting standards with 10,000 IU/mL, 1,000 IU/mL and 100 IU/mL. It is noteworthy that both systems demonstrated the highest intra-run CV% when detecting the 100 IU/mL standards, reaching 4.84% and 4.92% respectively. In contrast, while CAP/CTM maintained intra-run CV%<5% for standards with 10,000 IU/mL and 1,000 IU/mL, its CV% increased to 6.18% when detecting the 100 IU/mL standards, exceeding the 5% threshold (**Table 3**).

**Table 3:** Analysis of quantitative accuracy and precision of the three systems.

VL	Cobas 6800		Cobas 5800		CAP/CTM	
	Quantitative bias	Intra-run CV%	Quantitative bias	Intra-run CV%	Quantitative bias	Intra-run CV%
10,000 IU/mL	0.1 log	1.96%	-0.05 log	2.15%	-0.17 log	2.66%
1,000 IU/mL	0.02 log	3.22%	-0.02 log	2.50%	-0.22 log	3.23%
100 IU/mL	-0.03log	4.84%	0.16 log	4.92%	0.08 log	6.18%

#### 3.1.2 All three testing systems demonstrated excellent LOD

Probit regression analysis revealed that the LOD for cobas 6800, cobas 5800 and CAP/CTM were determined to be 24 IU/mL (95% CI: 17.839-74.960 IU/mL), 24 IU/mL (95% CI: 17.839-74.960 IU/mL) and 30 IU/mL (95% CI: 22.981-59.503 IU/mL), respectively, which were comparable to the LOD stated in the manufacturer’s instructions.

#### 3.1.3 All three testing systems exhibited good linearity within their quantitative ranges

**Figure 1:**
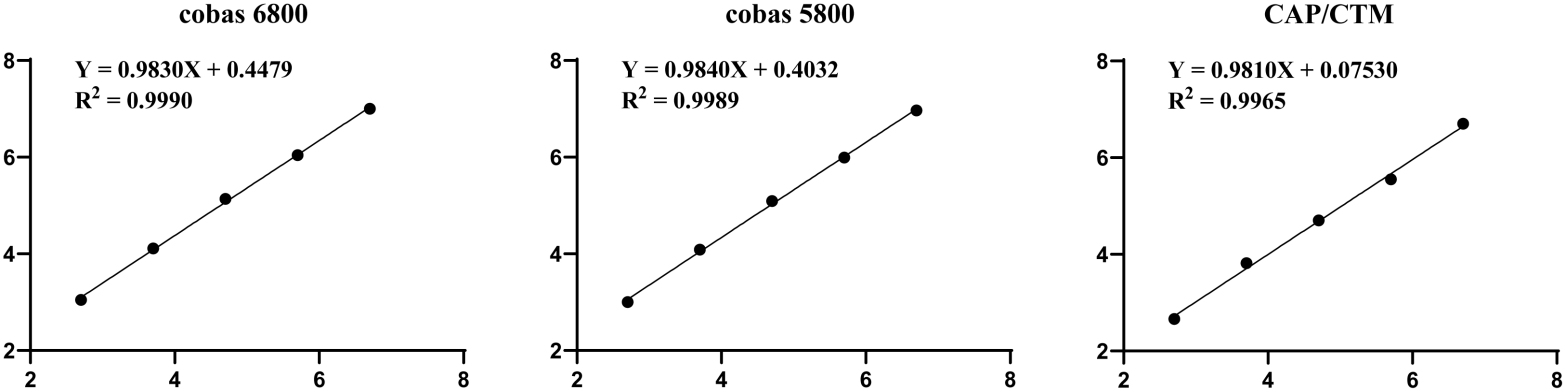
Linear regression plots of cobas 6800, cobas 5800 and CAP/CTM. Viral load measurements are plotted against expected values of serially diluted standards; solid lines denote fitted linear regression trends.

The linear regression coefficients for cobas 6800, cobas 5800 and CAP/CTM exceeded 0.99, were 0.9990, 0.9989 and 0.9965, respectively (**Figure 1**), demonstrating that all the testing system exhibited good linear performance across their quantitative measurement ranges.

#### 3.1.4 All three testing systems demonstrated favorable clinical performance

All three testing systems demonstrated 100% sensitivity and specificity, and were capable of detecting five prevalent HIV-1 subtypes in China, while yielding no false-positive results for the nine potentially interfering samples.

**Figure 2:**
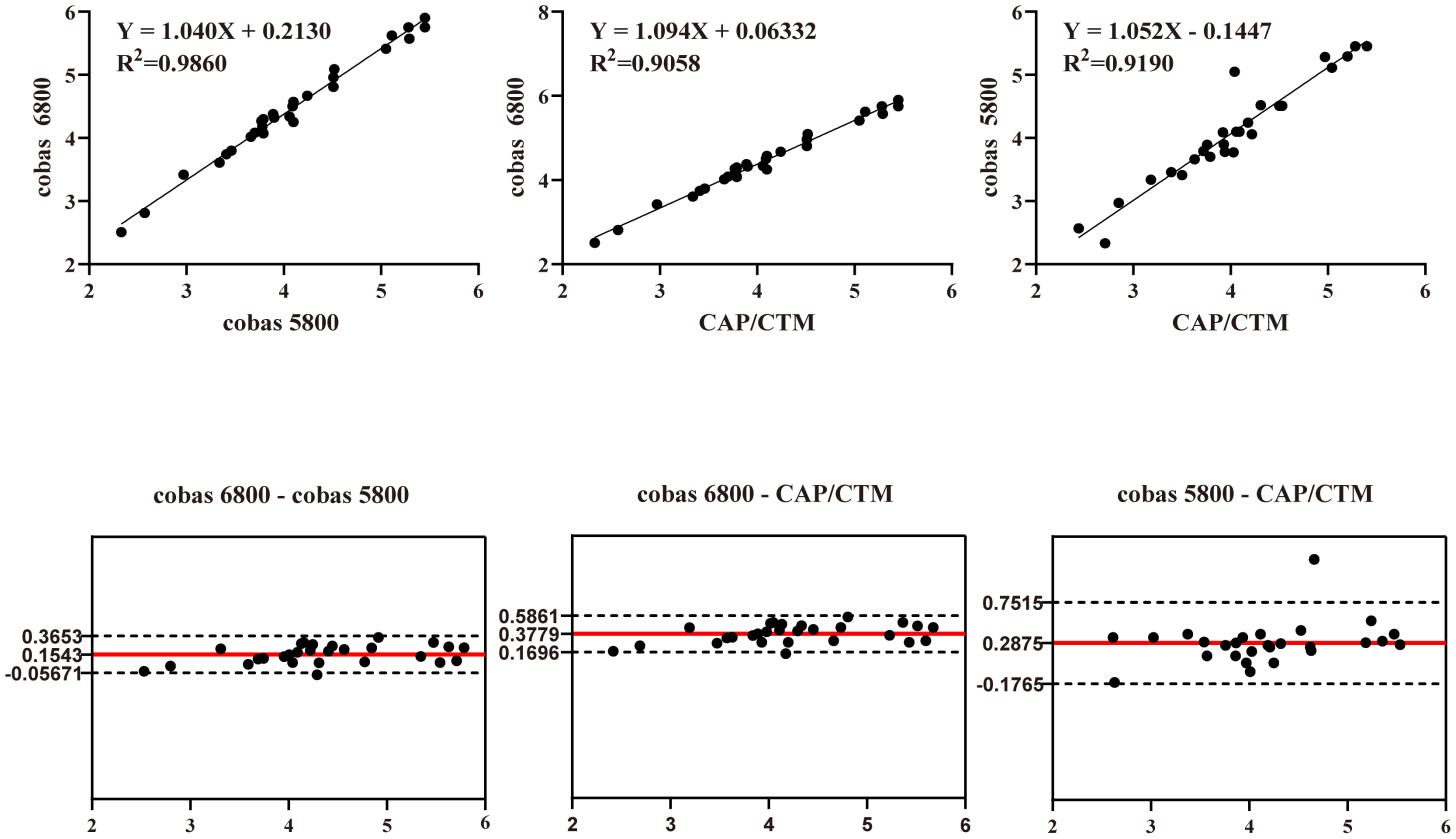
Correlation and Bland-Altman agreement analysis of quantitative results from the performance evaluation panel. Each black dot represents a positive sample; red solid lines indicate mean bias, and black dashed lines represent 95% limits of agreement (± 1.96 SD).

Pairwise comparisons of the three systems revealed strong correlation and agreement (**Table 4**, **Figure 2**). The highest correlation coefficient (R^2^) was observed between cobas 6800 and cobas 5800, reaching 0.9860, with the minimal bias of 0.1543 (6800 - 5800). For cobas 6800 and CAP/CTM, the R^2^ was 0.9058, and the bias was 0.3779 (6800 - CAP/CTM). Additionally, the R^2^ between cobas 5800 and CAP/CTM was 0.9190, with a bias of 0.2875 (5800 - CAP/CTM).

**Table 4:** Correlation and consistency analysis of performance evaluation panel.

Testing systems	Linear regression equation	R^2^	Bias
cobas 6800 and cobas 5800	y = 1.040x + 0.2130	0.9860	0.1543
cobas 6800 and CAP/CTM	y = 1.094x + 0.06332	0.9058	0.3779
cobas 5800 and CAP/CTM	y = 1.052x - 0.1447	0.9190	0.2875

### 3.2 Multi-center evaluation

#### 3.2.1 Detection rate of the multi-center evaluation panel

Testing results of the multi-center evaluation panel revealed that for samples with VL≥100 IU/mL, cobas 6800, cobas 5800 and CAP/CTM achieved a detection rate of 100% (all positive samples were correctly identified), and none of the negative samples produced a detectable result. When testing samples with VL<100 IU/mL, a certain percentage of samples were not detected due to factors such as Poisson distribution and multi-center sample pretreatment (**Table 5**). However, the overall detection rate of the cobas 5800 and cobas 6800 systems was higher than that of CAP/CTM.

**Table 5:** Detection of the multi-center evaluation panel.

Subtypes	VL (IU/mL)	Cobas 6800		Cobas 5800		CAP/CTM	
		Detection rate	CV%	Detection rate	CV%	Detection rate	CV%
CRF 01_AE	25	0/6	N/A	5/6	26.86%	4/6	12.00%
	50	5/6	17.41%	5/6	25.65%	4/6	12.59%
	60	6/6	15.47%	5/6	27.50%	5/6	7.57%
	100	6/6	15.60%	6/6	7.28%	6/6	13.77%
	400	6/6	3.59%	6/6	3.05%	6/6	6.00%
	2,200	6/6	3.12%	6/6	2.95%	6/6	4.70%
	14,000	6/6	2.81%	6/6	1.62%	6/6	1.30%
CRF 07_BC	25	2/6	46.34%	4/6	23.51%	1/6	NA
	60	6/6	13.57%	5/6	9.32%	5/6	10.66%
	70	5/6	22.36%	6/6	17.90%	6/6	10.11%
	100	6/6	13.57%	6/6	16.09%	6/6	10.50%
	350	6/6	6.32%	6/6	5.73%	6/6	6.64%
	1,500	6/6	4.17%	6/6	3.52%	6/6	5.05%
	8,000	6/6	2.11%	6/6	2.28%	6/6	1.63%
CRF 08_BC	25	3/6	11.94%	2/6	3.90%	2/6	4.76%
	75	6/6	7.02%	6/6	9.83%	5/6	13.59%
	80	6/6	15.09%	6/6	17.51%	5/6	9.70%
	100	6/6	7.40%	6/6	13.96%	6/6	8.61%
	400	6/6	3.59%	6/6	4.49%	6/6	4.53%
	1,500	6/6	3.50%	6/6	4.39%	6/6	1.84%
	9,000	6/6	2.10%	6/6	1.88%	6/6	1.72%
B	40	3/6	16.56%	6/6	26.68%	3/6	13.78%
	50	5/6	17.22%	5/6	15.35%	6/6	4.38%
	60	3/6	16.36%	6/6	9.09%	5/6	14.26%
	100	6/6	12.40%	6/6	5.22%	6/6	15.57%
	300	6/6	4.72%	6/6	3.00%	6/6	6.33%
	1,600	6/6	3.21%	6/6	4.71%	6/6	4.65
	11,000	6/6	2.14%	6/6	3.06%	6/6	2.78
CRF 55_01 B	30	3/6	5.35%	3/6	16.35%	1/6	NA
	70	6/6	6.81%	6/6	6.99%	5/6	22.22%
	90	6/6	7.34%	6/6	12.86%	6/6	11.93%
	120	6/6	11.24%	6/6	8.38%	6/6	23.61%
	300	6/6	6.22%	6/6	6.40%	6/6	7.65%
	1,500	6/6	3.38%	6/6	4.27%	6/6	2.82%
	7,000	6/6	1.98%	6/6	2.73%	6/6	2.98%

#### 3.2.2 Strong correlation and agreement was observed between each pair of the testing systems

**Figure 3:**
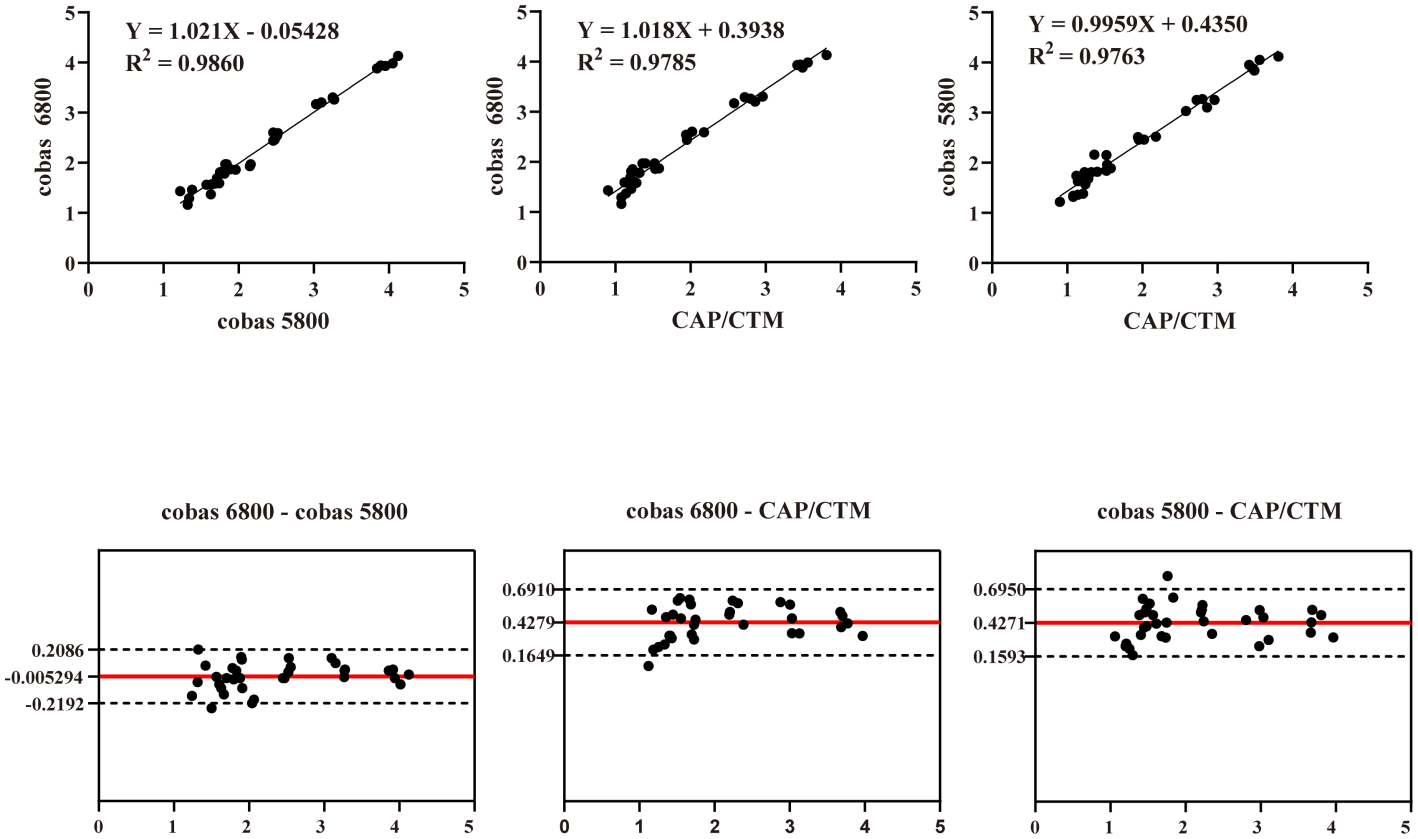
Correlation and Bland-Altman agreement analysis of quantitative results from the multi-center panel. Each black dot represents a positive sample; red solid lines indicate mean bias, and black dashed lines represent 95% limits of agreement (± 1.96 SD).

Quantitative results from each pair of the testing systems were compared using linear regression and Bland-Altman analysis (**Table 6**, **Figure 3**). Similar to the performance evaluation panel, the strongest correlation and the minimal bias was also observed between cobas 6800 and cobas 5800. The correlation equation, which represents the VL conversion relationship between the cobas 6800 and cobas 5800, was Y = 1.021X - 0.05428, with an R^2^ of 0.9860 and a bias of -0.005294 (6800 - 5800). Furthermore, the correlation equation between cobas 6800 and CAP/CTM was Y = 1.018X + 0.3938, and the R^2^ and bias was 0.9785 and 0.4279 (6800 - CAP/CTM), the correlation equation between cobas 5800 and CAP/CTM was Y = 0.9959X + 0.4350, with an R^2^ of 0.9763 and a bias of 0.4271 (5800 - CAP/CTM).

**Table 6:** Correlation and consistency analysis of multi-center evaluation panel.

Testing systems	Linear regression equation	R^2^	Bias
cobas 6800 and cobas 5800	y = 1.021x - 0.05428	0.9860	-0.005294
cobas 6800 and CAP/CTM	y = 1.018x + 0.3938	0.9785	0.4279
cobas 5800 and CAP/CTM	y = 0.9959x + 0.4350	0.9763	0.4271

#### 3.2.3 Improved inter-laboratory precision was observed as the VL increased across different subtypes

**Figure 4:**
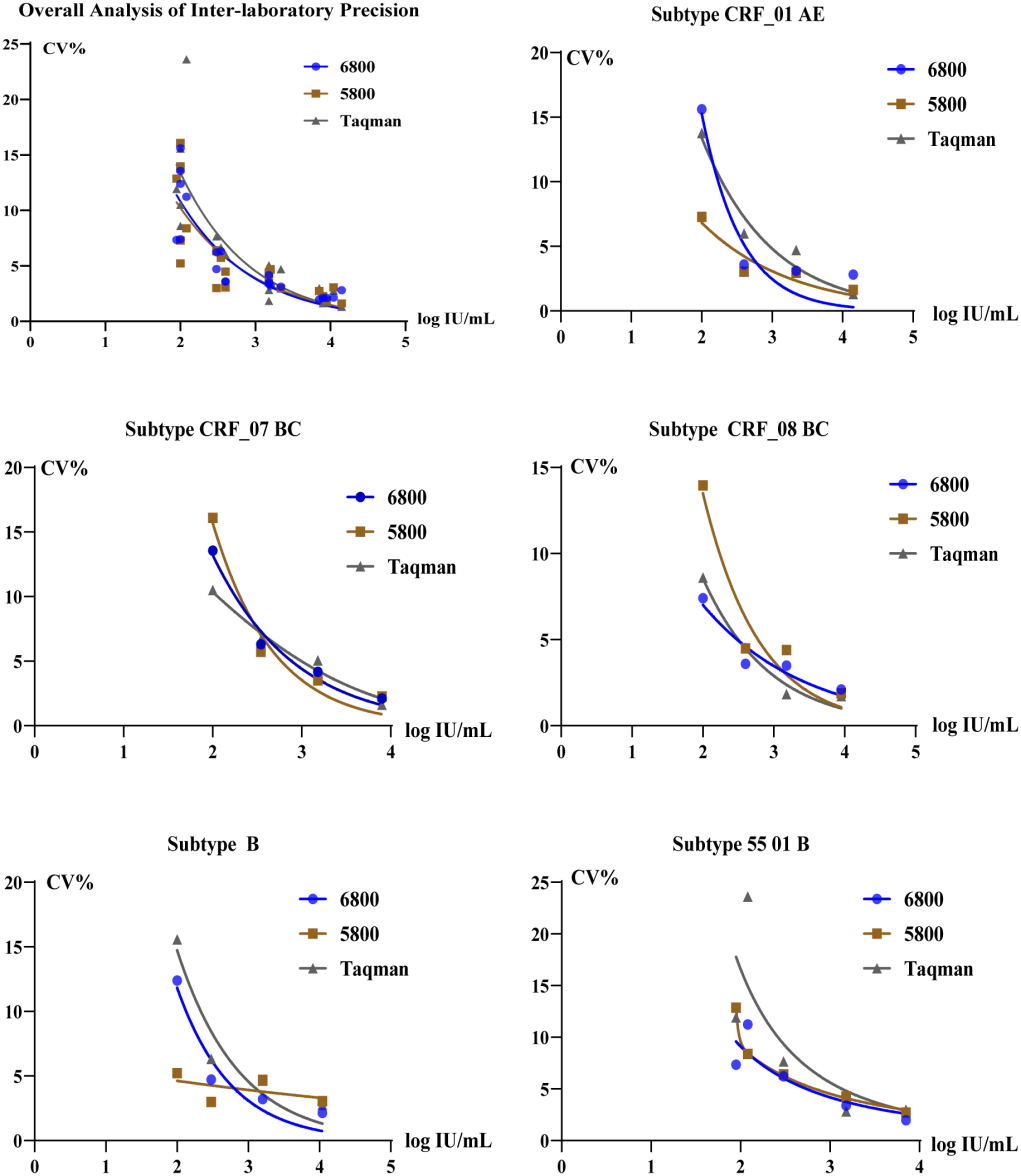
Inter-laboratory precision analysis of the multi-center panel. Each dot represents the inter-laboratory CV% for each testing system. The solid line represents the trend of inter-laboratory CV% for each testing system.

To minimize errors introduced by missing data, inter-laboratory precision analysis was performed only on samples with a detection rate≥95%. The analysis results indicate that for different HIV-1 subtypes, as the VL rises, all the three testing systems exhibited an increasing trend in inter-laboratory precision (**Table 5**, **Figure 4**), with CVs spanning from <2% at high concentrations to ~16% for low-level samples (100 IU/mL). The CAP/CTM platform exhibited the broadest performance range, recording both the lowest and highest overall variability depending on the viral load and genotype tested. At the commonly used concentration of 1,000 IU/mL in clinical performance verification, the inter-laboratory precision was all ≤5%.

#### 3.2.4 More HIV-1 RNA detected and quantified with the cobas 6800 and cobas 5800 system

**Figure 5:**
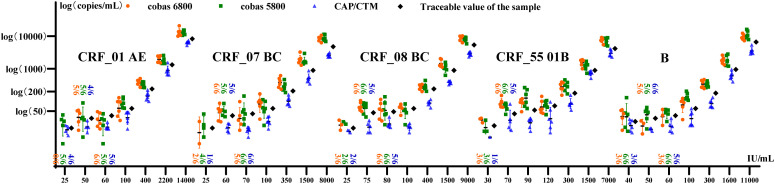
Distribution of quantitative results from the multi-center panel. Dots represent quantitative results from six laboratories; adjacent numbers indicate detection rates for low VL samples (<100 IU/mL); gray dots represent traceable values (1 copy/mL = 1.67 IU/mL).

By plotting scatter diagrams to analyze the distribution of quantitative results from the multi-center evaluation panel (**Figure 5**), the findings revealed that for different HIV-1 subtypes, as the VL rises, the dispersion of quantitative measurements from cobas 6800, cobas 5800 and CAP/CTM systems decreased. Moreover, it is evident that the quantitative results from cobas 6800 and cobas 5800 were closely aligned, yet both were higher than those obtained with the CAP/CTM system.

As indicated in the manufacturer’s instructions, the conversion factor between copies/mL and IU/mL for cobas 6800, cobas 5800 and CAP/CTM is uniformly set at 1 copy/mL = 1.67 IU/mL. When applying this conversion factor to establish the theoretical values for each traceable sample, it was observed that the quantitative results of cobas 6800, cobas 5800 and CAP/CTM all showed deviations within 0.5 log from the theoretical values.

### 3.3 Clinical application evaluation

#### 3.3.1 Switching the CAP/CTM testing system to the cobas 6800 may lead to increased detection of low VL samples

**Figure 6:**
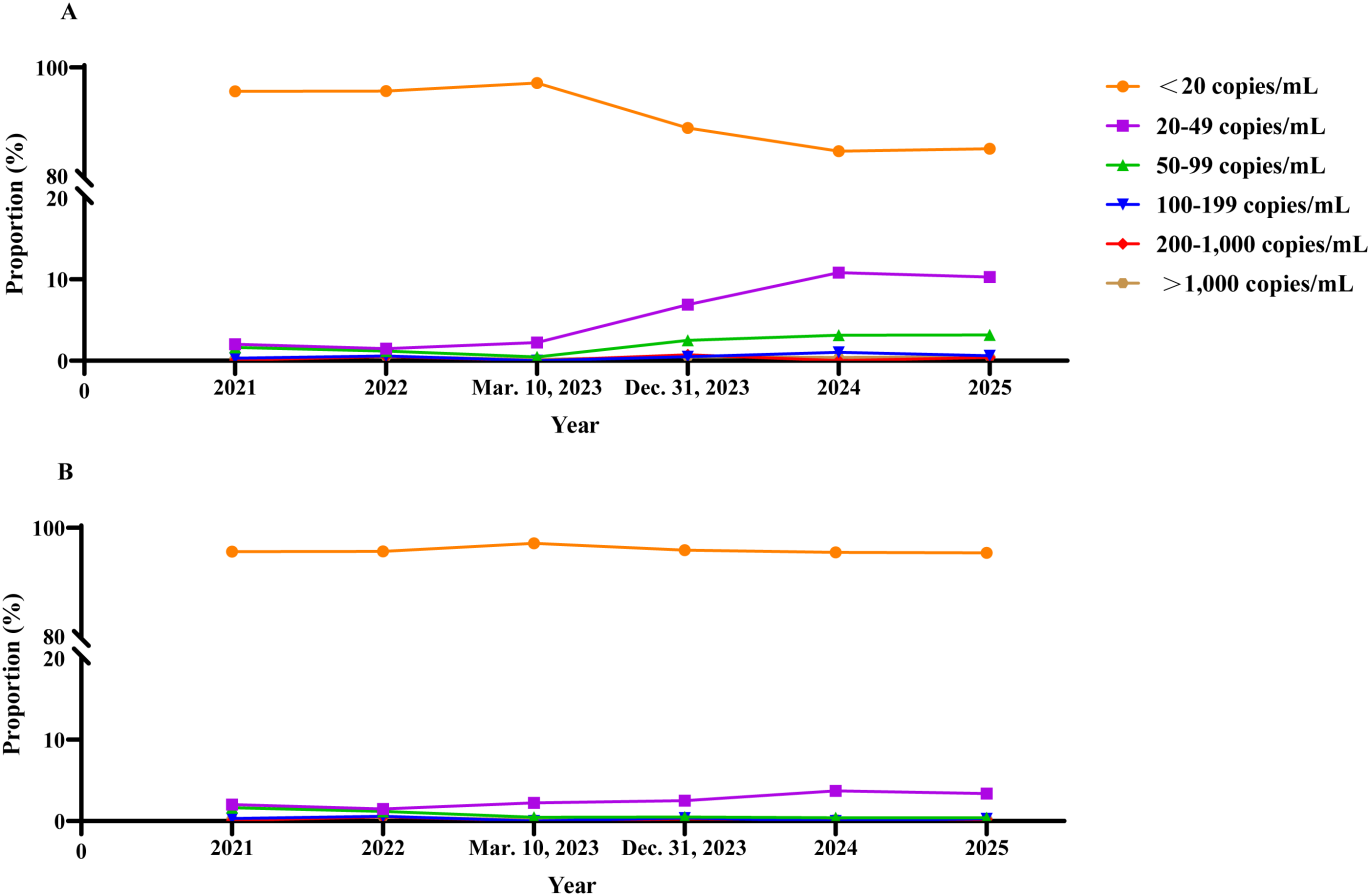
Analysis of VL distribution among 1,486 patients on consistent ART in Yunnan province during the period 2021 to 2025. **(A)** Crude VL distribution proportions; **(B)** VL distribution proportions after conversion using cobas 6800-CAP/CTM multi-center correlation coefficients.

Analysis of VL testing records from 1,486 patients in Yunnan Province who did not switch ART regimens between 2021 and 2025 revealed that, following the transition to the cobas 6800 testing system, the proportion of patients with a VL below 20 copies/mL significantly decreased by approximately 9.98% on average (**Table 7**, **Figure 6A**). Meanwhile, the mean proportion of patients with a VL between 20–49 copies/mL substantially increased by about 7.41%, those with a VL between 50–99 copies/mL increased by about 1.84%, and those with a VL between 100–199 copies/mL increased by approximately 0.41%. In contrast, no significant change was observed in the proportion of patients with a VL exceeding 200 copies/mL.

**Table 7:** Distribution of VL among 1,486 patients in Yunnan Province who did not switch ART regimens from 2021 to 2025.

Year	2021	2022	2023 (Jan. 1 - Mar. 10)	2023 (Mar.11 - Dec. 31)	2024	2025
Testing System	CAP/CTM	CAP/CTM	CAP/CTM	cobas 6800	cobas 6800	cobas 6800
With VL Results (N)	1,230	1,329	625	797	1,433	1,448
VL<20 copies/mL	95.61% (1,176/1,230)	95.64% (1,271/1,329)	97.12% (607/625)	88.84% (708/797)	84.58% (1,212/1,433)	85.01% (1,231/1,448)
20–49 copies/mL	2.04% (25/1,230)	1.50% (20/1,329)	2.24% (14/625)	6.90% (55/797)	10.82% (155/1,433)	10.29% (149/1,448)
50 - 99copies/mL	1.63% (20/1,230)	1.20% (16/1,329)	0.48% (3/625)	2.51% (20/797)	3.14% (45/1,433)	3.18% (46/1,448)
100–199 copies/mL	0.32% (4/1,230)	0.60% (8/1,329)	0% (0/625)	0.50% (4/797)	1.04% (15/1,433)	0.62% (9/1,448)
200 - 1,000 copies/mL	0.08% (1/1,230)	0.53% (7/1,329)	0% (0/625)	0.75% (6/797)	0% (0/1,433)	0.41% (6/1,448)
VL > 1,000 copies/mL	0.32% (4/1,230)	0.53% (7/1,329)	0.16% (1/625)	0.50% (4/797)	0.42% (6/1,433)	0.49% (7/1,448)

#### 3.3.2 Following conversion using cobas 6800-CAP/CTM multi-center correlation coefficients, patients showed consistent distribution across years

After converting the cobas 6800 test results using the correlation relationship between cobas 6800 and CAP/CTM established through the multi-center evaluation, the mean proportion of patients with a VL below 20 copies/mL was 95.56%, indicating that the difference from the mean proportion before March 10, 2023 decreased from 9.98% to 0.56% (**Table 8**; **Figure 6B**).

**Table 8:** Distribution among 1,486 patients in Yunnan Province after conversion based on the correlation relationship between cobas 6800 and CAP/CTM obtained from the multi-center evaluation.

Year	2021	2022	2023 (Jan. 1 - Mar. 10)	2023 (Mar.11 - Dec. 31)	2024	2025
Testing System	CAP/CTM	CAP/CTM	CAP/CTM	cobas 6800	cobas 6800	cobas 6800
With VL Results (N)	1,230	1,329	625	797	1,433	1,448
VL<20 copies/mL	95.61% (1,176/1,230)	95.64% (1,271/1,329)	97.12% (607/625)	95.86% (764/797)	95.46% (1368/1433)	95.37% (1381/1448)
20–49 copies/mL	2.04% (25/1,230)	1.50% (20/1,329)	2.24% (14/625)	2.51% (20/797)	3.70% (53/1433)	3.38% (49/1448)
50 - 99copies/mL	1.63% (20/1,230)	1.20% (16/1,329)	0.48% (3/625)	0.50% (4/797)	0.42% (6/1433)	0.41% (6/1448)
100–199 copies/mL	0.32% (4/1,230)	0.60% (8/1,329)	0% (0/625)	0.38% (3/797)	0% (0/1433)	0.28% (4/1448)
200 - 1,000 copies/mL	0.08% (1/1,230)	0.53% (7/1,329)	0% (0/265)	0.25% (2/797)	0.21% (3/1433)	0.21% (3/1448)
VL > 1,000 copies/mL	0.32% (4/1,230)	0.53% (7/1,329)	0.16% (1/625)	0.50% (4/797)	0.21% (3/1433)	0.35% (5/1448)

Similarly, the proportion of patients with a VL of 20–49 copies/mL decreased by an average of 6.14%, leaving it only 1.27% higher than the pre-March 10, 2023 levels. For patients in the 50–99 copies/mL range, the proportion decreased by 2.50%, bringing it within 0.66% of the historical mean. For patients with VL ≥100 copies/mL, no marked differences were observed, with proportions of 0.09%, 0.03%, and 0.02% across the respective subranges.

## 4 Discussion

The new-generation cobas 5800/6800 testing systems exhibit superior performance in detecting samples with low VLs compared to the CAP/CTM, as evidenced by enhanced sensitivity and improved precision for low-concentration standards. The slightly elevated CV% at 100 IU/mL for CAP/CTM remains within acceptable limits for a CV% approaching 20% is often observed and considered acceptable for measurements near the detection limits (Armbruster and Pry, 2008). Therefore, we conclude that the observed variation is likely attributable to inherent factors in low VL testing, such as Poisson distribution effects and sample homogeneity, which may naturally contribute to moderately elevated CV% (Luo et al., 2012; Figliozzi et al., 2016; Ikeno et al., 2018; He et al., 2022).

Consistent with previous studies (Wirden et al., 2017; Tan et al., 2018; Carmona et al., 2022; Tschäpe et al., 2022; Wirden et al., 2022), the present investigation demonstrated high correlation (R² > 0.90) between the quantitative results obtained from the new-generation cobas 5800/6800 systems and the conventional CAP/CTM system, although the new-generation systems yielded a modestly higher quantitative bias compared to CAP/CTM. This phenomenon was observed across both performance evaluation panels and multi-center panels. Overall, the cobas 5800 and 6800 systems demonstrated smaller quantitative biases compared to the CAP/CTM. The modestly higher results of the new systems are likely due to improved template extraction and amplification efficiency via technical optimizations (De Silva et al., 2025; Su et al., 2025). These improvements enable the detection of templates that were undetectable by the CAP/CTM, thereby providing more accurate, albeit higher, quantitative results.

Inter-laboratory precision analysis of the multi-center evaluation results revealed that all three testing systems achieved a 100% detection rate for samples with concentrations ≥ 100 IU/mL across all subtypes. However, reduced detection rates were observed for extremely low-concentration samples. Several factors may explain this phenomenon (Church et al., 2011; Anderson et al., 2025). First, at low concentrations, viral particles follow an inhomogeneous Poisson distribution, leading to increased dispersion due to the limited number of HIV-1 RNA copies; this may result in PCR templates that lack target molecules. Second, variations in the detection capabilities of the systems for different subtypes may account for this phenomenon, as the clinical samples in the multi-center panel included five HIV-1 subtypes prevalent in China (Vandenhende et al., 2015; Costa et al., 2024). Additionally, the multi-center evaluation introduced random errors associated with operators from different laboratories. Factors such as sample mixing, pipetting, and processing time at room temperature could contribute to variations in test results across laboratories, and these differences may become more pronounced when testing samples with low VLs. To minimize operational variations across different laboratories, future optimizations of testing systems may involve increasing the sample volume and template amount in amplification reactions. This approach would enhance HIV-1 RNA enrichment, thereby improving both detection rates and precision.

The clinical evaluation results indicated that for infected individuals receiving consistent treatment regimens, directly switching the testing system from CAP/CTM to cobas 6800 may increase the proportion of patients with VL between 20 and 200 copies/mL by 9.66%, leading to the erroneous reclassification as having LLV. In contrast, the proportion of VL above 200 copies/mL did not change significantly. Given that the treatment regimens for these patients remained unchanged, we believe these “slightly higher” VL values actually reflect a truer quantitative result due to the improved sensitivity of the testing system, rather than a decline in clinical control within the patient population. This artifact poses a significant challenge to monitoring the third 95% target, as a substantial proportion of patients may be erroneously categorized as not virologically suppressed, potentially triggering unnecessary clinical interventions or misdirected public health resource allocation. Importantly, the conversion equation derived in this study is not intended to replace individual clinical judgment or to be applied as a correction factor for routine patient results. Rather, it serves as a population-level calibration tool to facilitate consistent surveillance of viral suppression trends during the transition window, allowing public health authorities and clinical programs to interpret longitudinal data without artificial fluctuations caused by platform sensitivity differences. In this study, we found that after converting the cobas 6800 test results using the correlation relationship, no significant changes were observed in the proportions of infected individuals across different medical decision point ranges between 2021 and 2025, ensuring the continuity of treatment efficacy assessment during the transition from legacy to new testing systems. This also suggests that clinicians should consider the systematic bias introduced by the testing system itself after switching, especially when testing samples with low VL (Wirden et al., 2022; Costa et al., 2024).

We also noticed that the switch from the CAP/CTM testing system to the cobas 6800 primarily affected samples with VL below 200 copies/mL. For clinicians, the primary concerns are whether low-level viremia impacts clinical drug resistance and, in the long term, whether it influences clinical outcomes, including morbidity and mortality rates. While LLV (50–200 copies/mL) is associated with increased risk of virological failure, it has not been consistently linked to clinical events (Vandenhende et al., 2015; Simonetti, 2022; Bernal et al., 2025). Therefore, given the improved assay sensitivity and the systematic variability of results at LLV (Wang et al., 2025), both the appropriateness of setting the viral suppression threshold at 50 copies/mL and the optimal frequency of annual VL testing warrant further re-evaluation.

The study also existed some limitations. Firstly, when evaluating precision and accuracy, we did not strictly follow the guideline’s five-day requirement. To ensure comparability of the assessment results with previous results, we optimized the evaluation protocol for precision and accuracy. Specifically, we declined the assessment period from 5 days to 3 days. However, based on performance evaluation data and multi-center evaluation data, it is still demonstrate that the cobas 5800/6800 systems show higher precision and accuracy than CAP/CTM when testing samples at the hundreds of copies/mL level. Furthermore, as the cobas 5800 testing system had only just been introduced and put into use in China at the time of the study, a large number of clinical test results could not be obtained. Therefore, it was not possible to validate the conversion factors derived from the multi-center evaluation between the cobas 5800, cobas 6800, and CAP/CTM system in real clinical samples. Finally, the number of samples in the performance evaluation panel and multicenter evaluation panel may be relatively small; however, cross-validation of the data can help ensure the reliability of the results to a certain extent. Further in-depth exploration and evaluation with larger sample sizes are warranted in future studies.

## 5 Conclusion

The new-generation cobas 5800/6800 systems demonstrate superior analytical performance for HIV-1 subtypes prevalent in China, with enhanced sensitivity that more accurately reflects true virological status. While this advancement inevitably leads to increased detection of low-level viremia in previously suppressed patients, creating an apparent decline in viral suppression rates, this study provides a validated conversion approach to bridge the transition between legacy and new systems. By applying the multi-center derived correlation factor to clinical data from Yunnan Province, we demonstrate that the observed increase in LLV is attributable to analytical improvements rather than clinical deterioration. This framework enables healthcare systems to maintain accurate and comparable assessments of viral suppression efficacy during system transitions, thereby supporting the continued pursuit of the 95-95–95 targets.

## Data Availability

The raw data supporting the conclusions of this article will be made available by the authors, without undue reservation.
